# Driver licensing and reasons for delaying licensure among young adults ages 18-20, United States, 2012

**DOI:** 10.1186/2197-1714-1-4

**Published:** 2014-03-20

**Authors:** Brian C Tefft, Allan F Williams, Jurek G Grabowski

**Affiliations:** 1 AAA Foundation for Traffic Safety, 607 14th Street NW, Suite 201, Washington, District of Columbia 20005 USA; 2 Allan F. Williams LLC, 8200 Beech Tree Rd., Bethesda, Maryland 20817 USA

**Keywords:** Graduated driver licensing, Motor vehicle, Traffic

## Abstract

**Background:**

Motor vehicle crashes are the leading cause of death for teens and young adults in the United States. Graduated driver licensing (GDL) systems were designed to protect young novice drivers by limiting their exposure to specific risks while they gain experience driving. In the United States, most states’ GDL systems only apply to new drivers younger than 18. Some experts suggest that GDL might encourage young people to wait until age 18 to obtain a license, to avoid GDL requirements, resulting in older teenagers having less driving experience and higher crash risk than they might have had without GDL. This study examined the prevalence and timing of licensure among young adults, and explored factors associated with delaying licensure among those not licensed before age 18.

**Methods:**

An online questionnaire was completed by 1,039 persons aged 18-20 years, recruited from a representative panel of United States households. Main outcome measures were acquisition of driver’s license (a) within 12 months of the state minimum age for licensure, (b) before age 18. Associations of timing of licensure with demographic characteristics were assessed using multivariable logistic regression. Respondents not licensed before age 18 were asked to rate the importance of various possible reasons for delaying licensure.

**Results:**

54% of respondents were licensed before age 18. Blacks (37%; adjusted Prevalence Ratio 0.67, 95% Confidence Interval 0.48–0.93) and Hispanics (29%; adjusted Prevalence Ratio 0.60, 95% Confidence Interval 0.45–0.81) were less likely than non-Hispanic whites (67%) to be licensed before age 18. Lower household income was independently associated with delayed licensure (P < .001). The most common self-reported reasons for not becoming licensed sooner were not having a car, being able to get around without driving, and costs associated with driving.

**Conclusions:**

There was little evidence that GDL is a major contributor to delayed licensure; however, a substantial minority of young people do not obtain a driver’s license until age 18 or older and thus begin driving outside of the GDL system, which in most states only applies to new drivers younger than 18. More research is needed to investigate the safety of older novice drivers.

## Background

Motor vehicle crashes are the leading cause of death for children, teenagers, and young adults in the United States (US) (Subramanian 2012). Graduated driver licensing (GDL) systems, first introduced in the US in 1996, were designed to protect young novice drivers by restricting exposure to risk initially and then gradually phasing in increased privileges as the driver gains experience. While numerous studies have found that strong GDL programs are associated with lower fatal crash involvement rates for 16-year-olds (Williams et al. 2013; Zhu et al. 2013), some recent studies have found that they are also associated with higher rates for 18-year-olds (Masten et al. 2011; Fell et al. 2012). Some experts have suggested that GDL might encourage young people to wait until after their 18th birthday to obtain a license, to avoid requirements and restrictions that most states’ GDL systems only apply to new drivers younger than 18 (Masten et al. 2011).

There is little reliable data on the age at which young people typically obtain driver’s licenses and whether this changed significantly since the implementation of GDL systems. Because most states’ GDL systems only apply to new drivers younger than 18, data on the age at which new drivers typically begin driving could have important public health implications. If a substantial proportion of young people do not obtain driver’s licenses until they are 18 years old or older, this would imply that many new drivers are learning to drive without the benefit of GDL. The objectives of this study were to estimate the prevalence of delayed licensure among young people in the United States, and to investigate factors associated with and reasons for delayed licensure among those who delayed.

## Methods

### Participants

Young adults aged 18–20 years were sampled from an online research panel of households recruited using probability-based random digit dial telephone and address-based survey sampling methods. Households that lacked Internet access at the time of recruitment into the panel were provided Internet access and a laptop computer at no cost. Full documentation of panel recruitment and survey administration protocol are provided in GfK (2012).

The panel was pre-screened to identify households with at least one member aged 18-20 years. Invitations to complete an online questionnaire were e-mailed in June of 2012 to 1,464 panel members aged 18-20. Invitations were also sent to 2,082 older panel members known to be a parent of an adult child aged 18-20, to request their child’s participation in the study. Responses were obtained from 642 of the 18- to 20-year-olds contacted directly (completion rate 43% [Callegaro and DiSogra 2008). Of the 2,082 parents contacted, 1,107 (53%) returned a brief screener and forwarded the survey invitation to their adult child, 409 18- to 20-year-old children of these panel members (37%) completed the questionnaire, yielding a total of 1,051 respondents. Twelve respondents were identified as ineligible due to their age (age < 18 years or age ≥ 21 years) and were excluded. Three respondents were 20 years old when survey invitations were sent but turned 21 before they completed the questionnaire; they were included and were coded as 20 years old.

### Questionnaire

The questionnaire asked respondents to indicate whether they had a driver’s license, a learner’s permit, or neither. To confirm licensure status, respondents who indicated that they had a license were asked whether the license allowed them to drive without another adult in the vehicle. (Licensure status was re-coded to learner’s permit if the respondent indicated that the license did not allow driving without another adult in the vehicle.) Licensed respondents were asked to report the age in years and months at which they first obtained a license and the state in which it was issued. If the respondent did not remember or reported an implausible age (e.g., younger than the minimum age for licensure in the respondent’s state), the respondent was asked to place the age at which he or she was licensed into one of four categories (less than 6 months, 6 months – 1 year, 1 – 2 years, or more than 2 years from the minimum age for licensure in the respondent’s state), and this was used to calculate the range of ages at which the respondent could have been licensed. Respondents who reported that they held a learner permit at the time of the survey and respondents who reported that they obtained a license more than 12 months after their state’s minimum age for licensure were asked to report the age at which they obtained a learner permit.

Respondents not licensed before their 18th birthday (including those not yet licensed) were shown a list of several possible reasons why a person might delay obtaining a license and were asked to rate each as a *very important reason*, *somewhat important reason*, *minor reason*, or *not a reason* why they did not obtain their license sooner.

### Statistical analysis

The data were weighted to account for differences in probability of selection for recruitment into the panel, differences in panelists’ probability of selection for this survey, and to align the characteristics of the respondents to those of the population of US residents ages 18–20 with respect to age, sex, race and ethnicity, education, Census region, whether or not they lived in a metropolitan area, and number of household members aged 18-20.

Binary variables were derived to indicate whether the respondent was licensed (a) within 12 months of the minimum age for licensure in his or her state (Insurance Institute for Highway Safety Insurance Institute for Highway Safety 2013) and (b) before age 18. Missing values of these variables (n = 23) were multiply-imputed (Rubin 1987) using the method of chained equations (van Buuren et al. 1999; White et al. 2011). Twenty independent imputations were performed. Variables included in the imputation model were respondent age, sex, Census region (U.S. Census Bureau 2012), household income, self-reported race and ethnicity, self-reported urbanicity of the place where the respondent lived when he or she was 16 years old, and the minimum age for licensure in the state where the respondent obtained his or her license.

Multivariable logistic regression was used to estimate log-odds ratios for associations of each outcome variable (licensure within 12 months of state minimum age for licensure; licensure before age 18) with each demographic variable while adjusting for the other demographic variables. Marginal standardization was used to estimate adjusted probabilities of each outcome, which were then used to estimate adjusted prevalence ratios and 95% confidence intervals (Localio et al. 2007; Cummings 2011). All analyses were performed using Stata statistical software (StataCorp LP 2011), were based on weighted data, and took into account the variance associated with both the survey sampling method and the multiple imputation of missing values.

Analyses of self-reported reasons for delay in obtaining a license were descriptive only; no statistical tests were performed.

## Results

At the time of the survey, 70% of respondents reported that they had a driver’s license that allowed them to drive without another licensed adult in the vehicle, 12% reported that they had a learner’s permit, 1% reported having had a license or permit that had expired or had been suspended or revoked, and 17% reported having never obtained a driver’s license nor a learner’s permit (Table 1). The proportion of respondents who were licensed increased with age. Licensing rates of males and females were similar. Licensing rates were much higher in the Midwest than in other regions. The proportion of respondents who were licensed increased with increasing household income across all income categories. The proportion of respondents who were licensed was lower among those who self-identified as non-Hispanic black or Hispanic than among those who self-identified as non-Hispanic white. Respondents who reported that they lived “out in the country” when they were 16 years old were much more likely to have been licensed than respondents who lived in other areas.

**Table 1:** **Licensure status in relation to demographic characteristics**, **sample of 18**- **to 20**-**year**-**olds**, **United States**, **2012**

	Licensure status*		
	Driver’s license	Learner’s permit	Neither
	Row %		
All (n=1,039)	71	13	17
**Age** **(years)**			
18 (n=329)	65	18	17
19 (n=359)	70	13	16
20 (n=351)	76	7	16
**Sex**			
Male (n=468)	70	12	18
Female (n=571)	71	14	15
**Census region**			
Northeast (n=193)	64	13	22
Midwest (n=261)	82	12	6
South (n=316)	68	15	18
West (n=269)	71	10	20
**Place of residence at age 16** ^**†**^			
Out in the country (n=136)	79	14	7
Small town (n=212)	68	17	15
Medium-sized town (n=246)	72	12	17
Small city (n=224)	71	11	18
Large city (n=215)	68	11	22
**Household income**			
<$20,000 (n=200)	48	22	31
$20,000 – $39,999 (n=230)	53	15	32
$40,000 – $59,999 (n=159)	71	16	13
$60,000 – $99,999 (n=233)	82	10	8
$100,000+ (n=217)	88	7	5
**Race & ethnicity**			
Non-Hispanic white (n=632)	79	10	11
Non-Hispanic black (n=96)	55	23	22
Non-Hispanic other, incl. 2+ races (n=90)	75	15	11
Hispanic (n=221)	57	14	29

Notes: Row percents may not add to 100 due to rounding.

*6 respondents who reported that their drivers’ license had been suspended or revoked were counted as licensed, and 12 respondents who reported that their learner’s permit had expired or had been suspended or revoked were counted as having learner’s permits because the focus of the study was license acquisition.

^†^6 respondents with missing data on of place of residence at age 16 were excluded.

Substantial delay in licensure was observed: only 44% of respondents reported that they obtained driver’s license within 12 months of the minimum age for licensing in their state, and only 54% reported that they obtained a license before their 18th birthday (Table 2). Approximately half of respondents (52%) obtained a learner’s permit within 12 months of the minimum age to obtain a permit, and 72% obtained a permit before age 18.

**Table 2:** **Timing of driver licensure in relation to demographic characteristics**, **sample of 18**- **to 20**-**year**-**olds**, **United States**, **2012**

	Licensed within 12 months of state minimum age		Licensed before 18 ^th^ birthday	
	Unadjusted %	Adjusted prevalence ratio* (95% CI)	Unadjusted %	Adjusted prevalence ratio* (95% CI)
All (n = 1,039)	44		54	
**Age** **(years)**				
18 (n = 329)	45	1 [Reference]	56	1 [Reference]
19 (n = 359)	42	1.01 (0.80–1.27)	52	0.95 (0.79–1.15)
20 (n = 351)	45	1.09 (0.88–1.35)	53	1.02 (0.86–1.21)
**Sex**				
Male (n = 468)	42	1 [Reference]	51	1 [Reference]
Female (n = 571)	46	1.01 (0.84–1.21)	57	1.05 (0.91–1.22)
**Census region**				
Northeast (n = 193)	48	1 [Reference]	50	1 [Reference]
Midwest (n = 261)	56	1.21 (0.95–1.54)	68	1.48 (1.19–1.85)
South (n = 316)	38	1.01 (0.77–1.32)	51	1.30 (1.03–1.63)
West (n = 269)	39	0.98 (0.74–1.29)	49	1.23 (0.97–1.55)
**Place of residence at age 16** ^**†**^				
Out in the country (n = 136)	57	1 [Reference]	69	1 [Reference]
Small town (n = 212)	42	0.87 (0.63–1.20)	50	0.82 (0.64–1.05)
Medium-sized town (n = 246)	45	0.84 (0.62–1.15)	56	0.84 (0.66–1.07)
Small city (n = 224)	47	0.97 (0.71–1.33)	54	0.89 (0.70–1.14)
Large city (n = 215)	34	0.80 (0.56–1.14)	48	0.86 (0.67–1.11)
**Household income**				
<$20,000 (n = 200)	16	0.28 (0.17–0.47)	25	0.37 (0.25–0.55)
$20,000 – $39,999 (n = 230)	27	0.50 (0.36–0.71)	34	0.54 (0.41–0.70)
$40,000 – $59,999 (n = 159)	44	0.72 (0.54–0.95)	52	0.68 (0.54–0.85)
$60,000 – $99,999 (n = 233)	52	0.81 (0.65–1.01)	64	0.81 (0.68–0.96)
$100,000+ (n = 217)	67	1 [Reference]	79	1 [Reference]
**Race & ethnicity**				
Non-Hispanic white (n = 632)	56	1 [Reference]	67	1 [Reference]
Non-Hispanic black (n = 96)	24	0.57 (0.36–0.90)	37	0.67 (0.48–0.93)
Non-Hispanic other, incl. 2+ races (n = 90)	46	0.94 (0.69–1.28)	51	0.87 (0.67–1.13)
Hispanic (n = 221)	21	0.57 (0.38–0.87)	29	0.60 (0.45–0.81)

Notes: Missing values for timing of licensure (n = 23; 3% of weighted responses) were imputed 20 times and averaged.

*Adjusted prevalence ratios estimated using multivariable logistic regression followed by marginal standardization.

^†^6 respondents with missing data on place of residence at age 16 were excluded.

In multivariable analysis, the factor most strongly related to delay in licensure was household income: only 16% of respondents from households that reported annual incomes of less than $20,000 obtained their license within 12 months of their state’s minimum age for licensure, and 25% obtained their license before their 18th birthday. Among respondents from households that reported annual incomes of $100,000 or more, 67% obtained their license within 12 months of the minimum age and 79% did so before their 18th birthday (Table 2). Race and ethnicity were also significantly associated with the timing of licensure: the proportions of respondents licensed within 12 months of their state’s minimum age and prior to their 18th birthday were all much lower among respondents who self-identified as black or Hispanic than among those who self-identified as non-Hispanic white. Respondents from the Midwest were more likely to be licensed before their 18th birthday than respondents from other parts of the country. The proportions licensed within 12 months of the state minimum age and before age 18 did not vary significantly by age, sex, or level of urbanicity after other variables were controlled.

Among respondents who did not obtain their license before age 18, the reasons most frequently rated as somewhat or very important were: not having a car, ability to get around without driving, the cost of gasoline, the cost of driving overall, and just not getting around to it (Table 3). Fewer than one in four rated the difficulty associated with special requirements for younger drivers or not wanting a license with special restrictions for younger drivers as important reasons. Those who did not obtain a learner permit before age 18 were somewhat more likely to rate not wanting a license with special restrictions for younger drivers as an important reason for delaying licensure, compared with those who obtained a permit but not a license before age 18.

**Table 3:** **Reasons for delaying licensure among 18**- **to 20**-**year**-**olds not licensed before age 18**, **in relation to learner permit status at age 18**, **United States**, **2012**

	Learner permit before 18 ^th^birthday		All* (n = 458)
	Yes (n = 186)	No (n = 271)	
	% who rated item “Very important reason” or “Somewhat important reason” for not obtaining license sooner		
Did not have a car	33	52	44
Could get around without driving	31	45	39
Gas was too expensive	35	37	36
Driving was too expensive	38	34	36
Just didn’t get around to it	25	42	35
Could do what I wanted to do without driving	31	32	32
Was nervous about driving	24	34	30
Just not very interested in driving	27	30	29
Had to complete driver education course first	22	33	28
Getting a license was too expensive	27	26	26
Parents didn't have time to take me out to practice driving	21	28	25
Special requirements made it hard to get licensed at younger age	19	26	23
Too busy to spend time learning how to drive	18	23	21
Didn't want license with special restrictions that only applied to drivers under a certain age	14	24	21
Parents wouldn't let me get license sooner	24	17	20
To avoid having to take driver education	11	24	19
Could connect with friends online	15	19	17
Tried to obtain license sooner, but failed test	13	16	15
Took long time to get appointment for test	8	10	10

Notes: Missing values for timing of permit and licensure were imputed 20 times and averaged. Raw number (weighted %) of values imputed: License before 18^th^ birthday: 18 (2%); Permit before 18^th^ birthday: 63 (7%).

*Timing of permit was missing and could not be imputed for 1 driver in column “All”.

In post-hoc analysis of licensure among 19- and 20-year-old respondents not licensed before age 18, only 32% obtained a license before age 19 (Table 4). Of those who had obtained a permit but not a license before age 18, 59% obtained a license before age 19. In contrast, only 18% of those who did not obtain a permit before age 18 obtained a license before age 19, and only 38% even obtained a permit before age 19. Furthermore, of those not licensed before age 18, those who rated the difficulty of licensing requirements for younger drivers and/or not wanting a license with special restrictions for younger drivers as important reasons for delaying licensure were virtually no more likely to obtain a license before age 19 (32%) than were those who did not rate either item as important (30%).

**Table 4:** **Proportion of 19**- **and 20**-**year**-**olds licensed and proportion with learner permit before age 19 among those not licensed before age 18**, **United States**, **2012**

	Licensed before 19 ^th^birthday	Permit before 19 ^th^birthday
	% (*95*% *CI*)	
All (n = 332)	32 (24–40)	60 (52–68)
**Permit before 18** ^**th**^ **birthday**		
Yes (n = 125)	59 (46–72)	100 (–)
No (n = 207)	18 (10–26)	38 (28–48)

## Discussion

In a representative sample of young people, fewer than half obtained a driver’s license within a year of the minimum age for licensure in their state, and only slightly more than half obtained a driver’s license before their 18th birthday. At the time of the study, only one US state (New Jersey) applied a comprehensive GDL program to new drivers aged 18 years or older, and two (Maine and Maryland) applied some reduced GDL components to older new drivers. Although some young people may never drive, it is evident that at least a substantial minority of them obtain licenses without the protection that GDL is intended to provide to new drivers. If the proportion of respondents who had at least a learner’s permit at the time of the survey (84%) is treated as conservative estimate of the proportion of young people who will eventually obtain a license, this implies that 36% (95% CI 33%-39%) of those who will eventually obtain a license will do so after age 18 and thus outside of the GDL system.

Significant social and economic disparities were identified. Respondents who self-identified as black or Hispanic were significantly less likely to be licensed before age 18 compared with non-Hispanic whites, and lower household income was independently associated with decreased probability of licensure before age 18; these groups across the entire range of household incomes investigated. Only 32% (95% CI 25%-39%) of respondents who were black, Hispanic, or from households with annual incomes less than $20,000 were licensed before age 18; these groups accounted for 63% (95% CI 57%-69%) of all of those who were not licensed before age 18. If the proportion of these respondents who had at least a learner’s permit at the time of the survey (75%) is treated as a conservative estimate of the proportion of who will eventually obtain a license, this implies that 55% (95% CI 50%-61%) young people from these groups who obtain licenses will do so outside of the protective learning environment that GDL systems were designed to provide to new drivers.

The current popular perception is that young people are obtaining their driver’s licenses at older ages today than they did before GDL, and to a greater degree than would be explained by the GDL requirements themselves (e.g., requiring a new driver to hold a learner’s permit for a specified length of time before obtaining a license). However, studies conducted several years before the introduction of GDL systems in the US found that most young people did not obtain a license immediately upon reaching their state’s licensing age even before GDL. A study of data collected in 1983 found that fewer than 40% of young people in upstate New York, where the minimum age for licensure was 16, were licensed upon reaching age 17 (Williams et al. 1985). A national survey conducted in 1990 found that only 41% of 16-year-olds, 70% of 17-year-olds, and 77% of 18-year-olds were licensed (Federal Highway Administration 1991).

Shortcomings in multiple sources of data preclude ascertainment of changes over time in the specific ages at which most young people obtain licenses. Whereas a nationwide survey conducted in 1990 investigated licensure in a representative sample of driving-aged US residents (Federal Highway Administration 1991), similar surveys conducted in 1995, 2001, and 2008 only asked respondents whether or not they drove, but did not collect data on licensure (Federal Highway Administration 1997, 2004, 2011), prohibiting ascertainment of whether a person who reported driving held a license, a learner’s permit, or neither. Although studies of data reported by states to the US Department of Transportation appear to show a decreasing trend in licensing rates among teen-agers (Sivak and Schoettle 2012a), those data have been shown to contain substantial errors and thus are unusable for monitoring trends (Insurance Institute for Highway Safety 2006). A recent study of repeated cross-sectional surveys found that the proportion of high-school seniors who reported having a driver’s license declined from 85% in 1996 to 73% in Shults and Williams 1996–2010 (Shults and Williams 2013).

There has been concern that GDL has resulted in young people waiting until age 18 to begin driving in order to avoid the requirements and restrictions of GDL systems. While GDL has been shown to have reduced crash rates of 16-year-olds substantially and those of 17-year-olds moderately (Williams et al. 2013; Zhu et al. 2013, some recent studies have found that strong GDL systems have been associated with increased fatal crash involvement rates of 18-year-olds Masten et al. 2011; Fell et al. 2012). Some have suggested that this phenomenon may be due to young people delaying licensure until age 18 to avoid GDL requirements and restrictions (Masten et al. 2011). This study could not investigate that hypothesis directly, because GDL had already become virtually universal by the time participants in the current study had reached driving age. However, two results of this study suggest that desire to avoid GDL is at most a minor contributor to delayed licensure.

First, when young people not licensed prior to their 18th birthday were asked why they did not obtain their license sooner, the predominant reasons related to opportunity (e.g., not having a car), cost (e.g., gas was too expensive), and motivation (e.g., “could get around without driving”, “just didn’t get around to it”). Only 23% rated difficulties associated with the licensing system for younger drivers as an important reason, and only 21% rated not desiring a license with special restrictions for younger drivers as important.

Second, of 19- and 20-year-old drivers who had not obtained a license before their 18th birthday, fewer than one in three obtained a license before turning 19. Of those who had not even obtained a learner permit before their 18^th^ birthday, fewer than one in five obtained a license and fewer than two in five even obtained a permit before their 19^th^ birthday. If large numbers of young people were waiting until age 18 to obtain their license for the purpose of avoiding the GDL system, a larger proportion would have been expected to obtain licenses soon after turning 18 and a smaller proportion would have been expected to reach age 19 still not having a license.

One study of changes in licensing rates in several countries reported that higher rates of Internet access were associated with lower licensing rates of young people, and hypothesized that, “access to virtual contact reduces the need for actual contact among young people” (Sivak and Schoettle 2012b). The current study found minimal support for that hypothesis: only 17% of those not licensed before age 18 rated their ability to connect with friends online as an important reason why they did not obtain a license sooner.

### Limitations

This study relied on respondents to report accurately the age at which they obtained their learner permit and driver license. Some reported that they did not remember, and a few reported implausible ages which were treated as unknown. When age at licensure was unknown, respondents were asked to place it into one of a few broad categories relative to the minimum age for licensing in their state. This was used to determine whether the respondent was licensed within 12 months of the state’s minimum age for licensing and whether the respondent was licensed before age 18. These were imputed if they could not be determined from information provided by the respondent. Given the small number of respondents for whom licensing-related information was imputed (n = 23), imputed values had minimal impact on the overall results. The proportion of respondents licensed before age 18 was the same, 54%, whether based on all observations (including those with imputed data) or based on only observations in which age at licensure was known. It is also possible that some of the plausible responses could have been incorrect. However, we believe it is reasonable to assume that most respondents would have remembered whether they obtained their license before or after they turned 18, and thus that the main outcome reported here—only 54% of respondents obtained a driver’s license before their 18^th^ birthday—is unlikely to be biased by a large amount due to inaccurate reporting or missing data.

Results could also be biased if the timing of licensure were different among young people who completed our questionnaire than among the US population as a whole. Such bias could arise if young people who are members of the panel from which respondents were sampled were licensed earlier or later than the national average or if sampled panelists who completed the survey were licensed earlier or later than those who refused. The sample comprised both young people who were panelists themselves and young people who were not panelists but whose parents were. It seems unlikely that a young person’s licensure status would be related to one’s *parents*’ willingness to participate in surveys (and thus acceptance of the original invitation to join this survey research panel), and the timing of licensure was similar in both sampling frames (53% of respondents contacted directly and 55% of those contacted via their parents reported licensure before age 18), thus it appears unlikely that results would be biased due to differences between panel members and the general population with respect to timing of licensure. However, it is not possible to ascertain whether licensure rates differed between panel members who participated in our survey and those who were invited to participate but declined; results could be biased if the probability of responding to this survey (or to surveys in general) is related to licensure status.

This study was not able to identify specific reasons for differences observed in the timing of licensure in relation to demographic variables. For example, while respondents themselves identified cost as a barrier to earlier licensing, the reasons why race and ethnicity would be independently associated with licensing rates are not clear. While race/ethnicity and household income were correlated, differences in licensing rates by race/ethnicity persisted across all income strata (not shown). However, with our limited sample size, stratified analysis could not be performed across multiple variables simultaneously. It was surprising that differences in licensing rates in relation to urban versus rural residence—which were large in univariate analysis—were not statistically significant after controlling for other variables. It is possible that there truly are important differences in the timing of licensure between young people in urban versus rural areas, but that they were not detectable in our study due to the strong association of urban versus rural residence with race/ethnicity, associations of race/ethnicity with household income, and the associations of both race/ethnicity and household income with the timing of licensure.

Finally, although nationally representative, the results reported here do not necessarily reflect the situation in any given state. Substantial regional variation was observed. Because the sample was designed to be representative of the young adult population nationwide, 52% of all responses were obtained from only 9 states, which reflects the distribution of the US population.

## Conclusions

GDL systems were designed to protect new drivers; however, most states apply GDL only to new drivers younger than 18. A substantial minority of all young people—and a majority of those who are black, Hispanic, or from low-income households—do not obtain a driver’s license until they are 18 years old or older, and thus they begin driving outside of the GDL system. Little is known about the safety of older beginning drivers and the mechanisms by which they learn to drive. Given the size of this population, further research is needed to investigate the safety or risk of new drivers aged 18 years or older, to evaluate the potential impacts of extending GDL systems to this population, and to explore other ways to address the safety of older novice drivers.
